# Articular Doppler ultrasonography in juvenile idiopathic arthritis in remission: a prospective study

**DOI:** 10.1186/1546-0096-9-S1-P43

**Published:** 2011-09-14

**Authors:** Maria Teresa Terreri, Vanessa Bugni, Carolina Tavares, Sônia Mitraud, Rita NV Furtado, Jamil Natour, Maria Odete Hilário

**Affiliations:** 1 Department of Pediatrics, Pediatric Rheumatology, Universidade Federal de São Paulo/ Escola Paulista de Medicina, Brazil; 2 Department of Imaging Diagnostic, Universidade Federal de São Paulo/ Escola Paulista de Medicina, Brazil; 3 Division of Rheumatology, Universidade Federal de São Paulo/ Escola Paulista de Medicina, Brazil

## Background

Articular ultrasonography (US) in patients with juvenile idiopathic arthritis (JIA) in remission have shown subclinical synovitis. It is unclear whether these findings may predict subsequent joint damage and functional deterioration in asymptomatic joints.

## Aim

To assess the presence of subclinical synovitis by US in patients with JIA in remission and to evaluate its correlation with clinical and laboratory variables.

## Methods

Cross sectional study with prospective follow-up of 28 patients with JIA in remission. Inclusion criteria: oligoarticular or poliarticular subtypes, clinical/laboratory remission and age between 5-18 years. 12 healthy controls were evaluated at baseline. Clinical evaluation: physical examination, laboratory tests and ophthalmologic evaluation at baseline and every 6 months up to 24 months. Clinical assessment: active/ limited joint count; visual analogue scale (VAS) for each joint in patients older than 8 years, VAS of edema for each joint evaluated by a physician, systemic involvement, functional ability by the CHAQ, physician global VAS, patient global VAS, medications in use. Doppler US evaluation was performed in 17 joints bilaterally at baseline, at 12 and 24 months. US parameters: synovitis, synovial blood flow and bone erosion.

## Results

28 patients (22 girls, mean age 12.1 years), 21 oligoarticular and 7 polyarticular. 19 patients were in remission on medication and 9 off medication. In 952 joints evaluated, 68 showed thickening/limitation on physical examination. From the 57 altered US, 21 showed synovitis, 10 synovitis/positive flow, 3 synovitis/irregularities or erosions, 4 only positive flow and 19 only irregularities or erosions. Clinical assessment identified thickening in 49/895 joints that were normal on US. The US identified synovitis in 23/884 joints that were normal in clinical evaluation, characterizing subclinical synovitis. Concordance between clinical evaluation and US was observed in 90.9% joints. Prospective findings are still being assessed.

## Conclusion

Subclinical synovitis is present in patients with JIA in remission and may be related to prognosis and to predict relapse.

